# Understanding low chemoprevention uptake by women at high risk of breast cancer: findings from a qualitative inductive study of women’s risk-reduction experiences

**DOI:** 10.1186/s12905-021-01279-4

**Published:** 2021-04-16

**Authors:** Tasleem J. Padamsee, Megan Hils, Anna Muraveva

**Affiliations:** 1 Division of Health Services Management and Policy, College of Public Health, The Ohio State University, 280F Cunz Hall, 1841 Neil Avenue, Columbus, OH 43210 USA; 2 Lutheran Social Services of Central Ohio, Worthington, OH USA; 3 Division of Health Services Management and Policy, College of Public Health, The Ohio State University, Columbus, OH USA

**Keywords:** Breast cancer, Cancer prevention, Chemoprevention, Risk-reduction, Health information, Women’s health

## Abstract

**Background:**

Chemoprevention is one of several methods that have been developed to help high-risk women reduce their risk of breast cancer. Reasons for the low uptake of chemoprevention are poorly understood. This paper seeks a deeper understanding of this phenomenon by drawing on women’s own narratives about their awareness of chemoprevention and their risk-related experiences.

**Methods:**

This research is based on a parent project that included fifty in-depth, semi-structured interviews with a purposive sample of African American and White women at elevated risk of breast cancer. This specific study draws on the forty-seven interviews conducted with women at high or severe risk of breast cancer, all of whom are eligible to use chemoprevention for breast cancer risk-reduction. Interviews were analyzed using grounded theory methods.

**Results:**

Forty-five percent of participants, and only 21% of African American participants, were aware of chemoprevention options. Women who had seen specialists were more likely to be aware, particularly if they had ongoing specialist access. Aware and unaware women relied on different types of sources for prevention-related information. Those whose main source of information was a healthcare provider were more likely to know about chemoprevention. Aware women used more nuanced information gathering strategies and worried more about cancer. Women simultaneously considered all risk-reduction options they knew about. Those who knew about chemoprevention but were reluctant to use it felt this way for multiple reasons, having to do with potential side effects, perceived extreme-ness of the intervention, similarity to chemotherapy, unknown information about chemoprevention, and reluctance to take medications in general.

**Conclusions:**

Lack of chemoprevention awareness is a critical gap in women’s ability to make health-protective choices. Future research in this field must consider complexities in both women’s perspectives on chemoprevention and the reasons they are reluctant to use it.

**Supplementary Information:**

The online version contains supplementary material available at 10.1186/s12905-021-01279-4.

## Background

For many women at high risk of breast cancer due to family history or known genetic mutation, managing that risk is a key psychological concern. Such women face a lifetime breast cancer risk of 20–80%, and are often motivated by fear of cancer to search out ways to reduce this risk [1–3]. Chemoprevention is one of several risk-management methods developed for such women, and has been shown to decrease lifetime risk by approximately 50% for most subgroups of the high-risk population [4–7]. Chemoprevention involves taking a 5-year course of daily antiestrogen pills (usually tamoxifen for pre-menopausal women, raloxifene for post-menopausal women) as an alternative to surgical prevention options (bilateral prophylactic mastectomy and/or oophorectomy) [8], but uptake remains very low. The proportion of high-risk women eligible for primary prevention who take antiestrogen medication is 5% or less, and these numbers are only slightly higher for carriers of pathogenic BRCA variants [9–12].

Reasons for low use of chemoprevention are not yet fully understood, but prior studies have illuminated several important factors associated with low uptake of preventive medication or the rarer choice to use chemoprevention. One of the most important influences on these decisions is a healthcare provider’s recommendation for or against chemoprevention [11, 13–17]; the recommendation of a medical oncologist seems particularly strongly associated with use of chemoprevention [18, 19]. A variety of provider-level barriers also impede chemoprevention usage, however, including lack of confidence in using risk-prediction models to identify high-risk patients, and lack of confidence discussing risk or prescribing chemoprevention medications [20–23]. From the patient side of the equation, cancer worry/anxiety, perceived breast cancer risk, and objective cancer risk are positively associated with the use of chemoprevention [1, 11, 13, 15–19, 24–28], while underestimation of benefits and/or overestimation of risks, perception of (or worry about) low drug efficacy, and concerns about side effects are negatively associated with chemoprevention use [11, 20, 21, 27, 29–33]. Decision aids may increase women’s understanding of the risks and benefits of chemoprevention, but may not be consistently associated with either higher or lower uptake [15, 34, 35].

The mechanisms of these associations, however, are underexplored. Study designs have usually involved explicitly educating women about chemoprevention within the context of the study. It is therefore unclear whether correlates of uptake affect women’s general engagement in breast cancer prevention, their likelihood of choosing to use chemoprevention when that specific option is offered, or their likelihood of choosing chemoprevention over other risk-management options [36]. In addition, while lack of awareness and insufficient knowledge are among the top known barriers to chemoprevention uptake [14, 30, 37], few studies have examined how women who are aware of chemoprevention differ from those who are not. The vast majority of chemoprevention studies have also relied on samples of predominantly White participants, underrepresenting or nearly excluding women of other racial/ethnic backgrounds [24, 33, 34, 38]. Awareness of this problem has begun to cause a shift, with a few studies beginning to enroll more multi-racial participant groups [19, 25, 35, 39].

The current paper takes advantage of data related to chemoprevention choices obtained from a parent study designed to use high-risk women’s own stories about coping with risk to understand the dynamics of women’s prevention decision making. The parent study generated 50 open-ended qualitative interviews with White and African American women at elevated risk of breast cancer. In this paper, we draw on these detailed narratives to step beyond the context of a chemoprevention trial and better understand the factors that shape women’s awareness of and choices about chemoprevention.

## Methods

For the parent project upon which this paper is based, fifty semi-structured, in-depth interviews were conducted by the first author (Principal Investigator) between May 2015 and March 2016. The relevant interview protocol was developed specifically for this study (see Additional file 1 [40]). Participants were recruited from the High Risk Breast Cancer Program and Clinical Genetics Clinic at The Ohio State University (OSU) Comprehensive Cancer Center, and through the ResearchMatch national research volunteer database and Study Search online study listing tool at OSU. Eligible participants were at least 18 years old with an elevated risk of breast cancer but no personal history of cancer [41]. Interviews were conducted in-person or via telephone and lasted 22–120 min. Iterative data analyses (described below) occurred alongside data collection, and the decision to stop conducting interviews was made once it was clear that saturated information had been collected about all the core emergent interview themes [42]. Ethical approval was granted by the Institutional Review Board of The James Cancer Hospital, OSU Comprehensive Cancer Center, and the Ohio State University (Protocol # 2014C0101), and participants provided informed consent. Interviews covered a range of topics, including perceived breast cancer risk; sources and content of risk information; understanding and consideration of prevention options; decision-making processes and networks; and psychosocial well-being. Other findings from the parent project have been published and are under review elsewhere [3, 43–45].

For the specific study presented in this paper, analyses were limited to the 47 participants whose breast cancer risk was sufficiently high to render them eligible to use chemoprevention. This was an observational study of women’s experiences and perspectives; no participants were offered chemoprevention in the course of the study. Thirteen percent of participants (6) were categorized at “severe” lifetime risk of breast cancer (diagnosed with a pathogenic BRCA variant) and 87% (41) were categorized at “high” risk (family history including multiple, young, and/or bilateral cases of breast or ovarian cancer) [41]. Women in these categories have a quantitative lifetime risk of breast cancer over 20%; clinical guidelines published by the National Comprehensive Cancer Network (NCCN) recommend that healthcare providers discuss chemoprevention with such women.

Transcribed data were checked by the first author for accuracy and then analyzed in five stages, using a constructivist grounded theory approach [42]. First, three separate coders used open coding to generate exhaustive lists of themes present in the first eight interviews; these lists were compared to generate one comprehensive list of emergent themes. Some themes were anticipated by original interview questions and others emerged inductively from women’s own accounts. Related themes were then organized into categories. Second, each interview was coded systematically by at least two coders, who assigned segments of women’s stories to as many themes and categories as relevant. NVivo 11 (qualitative data management software) was used throughout this and the following stage of analysis. Additional themes and categories emerged during systematic exploration of the data. Coders caught one another’s omissions and discussed any areas of disagreement until reliable coding principles were agreed upon. Transcription, coding, and memo-ing stages all began while interviews were still being conducted, so that insights gained from analysis of early interviews could be reflected in the content of later interviews. By the end of data collection, all themes were theoretically saturated [42, 46].

The third stage of analysis involved in-depth exploration of themes and categories created in previous stages, using Excel tables that mapped relationships among themes, categories, and groups of participants, as well as memos that explored the content and boundaries of themes and categories. One important distinction that emerged from this process was between those who were ‘aware’ and ‘unaware’ of chemoprevention—being ‘aware’ was identified as having expressed any pre-interview understanding (regardless of terminology used) that medications exist that can reduce breast cancer risk among high-risk women. We therefore sorted participants into aware and unaware groups, and then examined those two groups further to compare them with respect to other emergent themes and distinctions that had emerged from inductive analysis. These themes included: exposure to breast cancer of a loved one; cancer worry; race; financial issues and SES; the presence of comorbid conditions; interactions with healthcare professionals; preferences toward and experiences with gathering information; body image and sexuality; and perceptions of risk and health beliefs. Finally, we sorted women who had heard of chemoprevention into five emergent categories of reflecting their “disposition toward chemoprevention,” and explored the relationships among themes that might influence women’s dispositions. These disposition categories were: (1) chose it; (2) leaning or leaned toward it; (3) aware and considering, or hasn’t decided yet; (4) aware and would consider under certain conditions; and (5) aware and would not consider, not considering, wouldn’t do, or considered and ruled out.

The first author was involved in all parts of the project: study design, data collection, and all stages of analysis and theory building. This consistent engagement ensured the continuous presence of a high level of expertise relevant to both the substance and the methodology of the study. The inclusion of two additional research team members throughout the coding, analysis, and write-up phases created important opportunities for the introduction of alternative perspectives and interrogation of assumptions that may have been made by a single author [47].

## Results

Participants’ ages ranged from 21 to 69, with a mean of 44 years. This study intentionally recruited a sample for which racial comparisons could be made within the constraints of the attainable sample size; 40% (19) of participants identified as African American and 60% (28) were non-Hispanic Whites. The sample consisted mostly of well-educated women—64% had at least a college degree. Half (51%) of participants were categorized as high socioeconomic status (SES) and the other half as medium/low SES, based on composite information including educational level, occupational status, and household income. See Table 1 for sample demographics. Participants are identified when quoted below by pseudonym, race, and age range; the latter groups are as follows: early 20s = 20–24; late 20s = 25–29; early 30s = 30–34; late 30s = 35–39; etc.

**Table 1:** Sample demographics

	African American	White	Total
SES			
Low	3	1	4 (9%)
Medium	9	10	19 (40%)
High	7	17	24 (51%)
Ashkenazi Jewish			
Yes	0	4	4 (9%)
No	19	24	43 (91%)
Age			
≤ 25	2	4	6 (13%)
26–35	5	3	8 (17%)
36–45	4	7	11 (23%)
46–55	2	4	6 (13%)
56–70	6	10	16 (34%)
Severity of risk			
Severe	0	6	6 (13%)
High	19	22	41 (87%)
Total	19 (40%)	28 (60%)	47 (100%)

Uptake of chemoprevention in this sample echoed findings of previous studies [48]. Of 47 women interviewed, only four had ever seriously considered chemoprevention; three of these women actually chose chemoprevention and took the prescribed medication. The inductive analyses described above generated insights about chemoprevention awareness as a necessary prerequisite for use of this risk-reduction method, about information access and personal characteristics associated with chemoprevention awareness, and about the concerns of women who are aware of chemoprevention but not inclined to consider it.

### Awareness of chemoprevention

Among our sample of women at elevated risk, fewer than half (21, or 45%) had ever heard of chemoprevention. Furthermore, awareness of chemoprevention was much more common among White participants, with 61% of White women and only 21% of African American women having heard of chemoprevention before the interview. Among ‘aware’ participants, the topic of chemoprevention came up in the context of women’s stories about conversations with healthcare providers about their breast cancer risk. After finally being persuaded by her family and healthcare providers to undergo genetic testing, Lucy recalled:

When I first found out I was *BRCA2* positive, I remember the genetic counselor went through all of my options: “We can do a prophylactic double mastectomy. You can have a hysterectomy. And we can put you on Tamoxifen…And it can be everything from that to we do nothing!” (White, late 40s)

In the course of remembering how she came to think of herself as having high risk for breast cancer, Molly explained:

It was when I had a mammogram that was suspicious and then they did a…dye ultrasound, and that was when I met Dr. Tong. And once I started telling my history and so forth, and we started really talking about it, that she placed me as a high risk. And then I participated in the STAR study (chemoprevention trial). (White, late 60s)

In contrast, chemoprevention never came up in the stories told by ‘unaware’ participants, who often reacted to the idea with surprise when the interviewer raised it late in the interview.

Q: So nobody's ever said anything about any medications that you might take?

A: To prevent cancer?

Q: Yeah.

A: Oh, heavens, no. Is there something out there? (Tanya, African American, early 50s)

I actually wasn't aware of the preventive drug regimens [before this interview]. This is a new thing for me. (Tara, White, early 30s)

Fewer participants had heard of chemoprevention than any of the other available risk-management tools: all participants knew about basic surveillance methods (specifically, mammograms and breast self-exams), 94% (44) knew about genetic testing, 83% (39) had heard of prophylactic mastectomy, and 51% (24) had heard of prophylactic oophorectomy. Along similar lines, we found that women who knew about surgical prevention options were also more likely to be aware of chemoprevention (Table 2): of the 7 women who had never heard of surgical prevention options none new about chemoprevention either, while 53% of the 40 women who knew of *at least one* surgical option and 64% of the 22 women who knew about *both* surgical options were aware of chemoprevention as well.

**Table 2:** Awareness of Chemoprevention and Surgical Risk-Reduction Options

	Aware of chemoprevention	Unaware of chemoprevention	Total^a^
Knows of no surgical options^b^	0 (0%)	7 (100%)	7 (15%)
Knows of at least one surgical option^b^	21 (53%)	19 (47%)	40 (85%)
Knows of both surgical options^b^	14 (64%)	8 (36%)	22 (47%)
Total^b^	21 (45%)	26 (55%)	47 (100%)

All categories coded inductively from qualitative data. For example, a woman was coded as “knows of no surgical options” if her entire narrative about how she has learned, thought, and decided about breast cancer prevention included no mention of surgical options, and she confirms not knowing about them when asked directly late in the interview

^a^Numbers in this column are row totals. Percentages are out of the sample of 47 participants analyzed in this paper

^b^Percentages on this row refer to the percent of the row total that falls in this column (e.g. 53% of those who know of at least one surgical option are aware of chemoprevention)

### Risk-reduction information

Exploring emergent themes that could be causally associated with chemoprevention awareness revealed a substantial role for mechanisms related to risk-reduction information. Most importantly, women’s awareness of chemoprevention appears to be related to their access to breast specialists, cancer specialists, and genetic counselors. It was often in conversations with these specialists that women were first exposed to specific risk-reducing options—including chemoprevention. This is reflected in Lucy’s experience with her genetic counselor and Molly’s experience with her breast oncologist, quoted above. Having an *ongoing* relationship (more than one appointment) with a specific specialist may make it even more likely that high-risk women have an opportunity to encounter information about chemoprevention. Teresa (African American, late 40s), for instance, began seeing a surgical oncologist for high-risk care when her mother was dying of breast cancer, then learned about and started a regimen of Tamoxifen through the course of regular appointments with her. Of the 26 women in our sample who had never seen a specialist only 27% had heard of chemoprevention, while 67% of the 21 women who had seen a specialist at least once and 71% of the 14 women who had ongoing contact with an individual specialist were aware of the chemoprevention option (Table 3).

**Table 3:** Awareness of Chemoprevention and Access to Risk-Reduction Information

	Aware of chemoprevention	Unaware of chemoprevention	Total^a^
Has never seen a relevant specialist^b^	7 (27%)	19 (73%)	26 (55%)
Has seen at least one relevant specialist^b^	14 (67%)	7 (33%)	21 (45%)
Has ongoing contact with at least one specialist^b^	10 (71%)	4 (29%)	14 (30%)

Main source of risk-related information is a healthcare provider^b^	13 (65%)	7 (35%)	20 (43%)
Main source of risk-related information is not a healthcare provider^b^	8 (30%)	19 (70%)	27 (57%)

Has discussed risk-reduction with a healthcare provider^b^	11 (85%)	2 (15%)	13 (28%)
Has never discussed risk-reduction with a healthcare provider^b^	10 (29%)	24 (71%)	34 (72%)
Total^b^	21 (45%)	26 (55%)	47 (100%)

All categories coded inductively from qualitative data. For example, a woman was coded as “has not seen a relevant specialist” if her entire narrative about how she has learned, thought, and decided about breast cancer prevention includes no mention of ever having seen a breast specialist, oncologist, or genetic specialist about anything related to her breast cancer risk

^a^Numbers in this column are row totals. Percentages are out of the sample of 47 participants analyzed in this paper

^b^Percentages on this row refer to the percent of the row total that falls in this column (e.g. 67% of those who have seen at least one relevant specialist are aware of chemoprevention)

While specialists seemed to play a particular role in informing women of the chemoprevention option, it was also more generally true that more participants who obtained information related to their breast cancer risk directly from any healthcare provider were aware of the chemoprevention option (Table 3). Of the 20 women who talked most often about a healthcare provider when discussing their sources of risk-related information, 65% were aware of chemoprevention—compared to only 30% of those who cited a different primary source of information. Similarly, women whose relationships with healthcare providers included at least one conversation explicitly about breast cancer risk-reduction were more likely to know of the chemoprevention option; 85% of women who had ever had this kind of conversation had heard of chemoprevention, compared to fewer than a third of women who hadn’t had such a discussion.

Beyond their healthcare providers, most women also have a much broader information environment. Participants described many sources of risk- and prevention-related information: healthcare providers, family members and friends, online research, their own medical knowledge, medical journals, newspapers and magazines, support groups, and others. While the *number* of information sources a woman used did not have a large impact on her prevention knowledge, we found that the *type* of information source played an important role. Women who were aware of chemoprevention were more likely to list healthcare providers, individuals with similar experiences, family members with medical or professional expertise, and high-risk support groups among their sources of information. Those unaware of chemoprevention were more likely to list magazines and newspapers or the experiences of family and friends.

### Personal characteristics

Two aspects of women’s personal characteristics also seemed to be meaningfully related to their chemoprevention awareness. First, those who were aware of chemoprevention often described a more nuanced information gathering approach than others. The nuanced approaches women described included being informed enough to ask the right questions (e.g. “I need to know what I need to know”); trying to validate information they received with a second source (e.g. seeking a second medical opinion or looking up peer-reviewed research online); being aware of information-seeking gaps (e.g. not knowing where to go for information); or having encountered challenges to gathering needed information (e.g. realizing their healthcare provider is not up-to-date on recommendations for managing high breast cancer risk). Lainie (White, late 50s), for instance, engaged in almost all of these strategies—seeking out multiple healthcare providers to fill in gaps in risk-related information, expressing frustration about not knowing who to ask for some types of information, asking her cousin for genealogy information and then taking this to a genetic counselor, asking for references to research papers, and more. Overall, 62% of participants who engaged in any of these nuanced approaches to information knew of chemoprevention, compared to only 31% of those who did not (Table 4).

**Table 4:** Awareness of chemoprevention and personal characteristics

	Aware of chemoprevention	Unaware of chemoprevention	Total^a^
Reports nuanced information gathering^b^	13 (62%)	8 (38%)	21 (45%)
Does not report nuanced information gathering^b^	8 (31%)	18 (69%)	26 (55%)
Low cancer worry^b^	6 (25%)	18 (75%)	24 (51%)
Moderate or high cancer worry^b^	15 (65%)	8 (35%)	23 (49%)

All categories coded inductively from qualitative data. For example, a woman was coded as “reports nuanced information gathering” if she discussed any of these strategies during her interview: trying to be informed enough to ask the right questions, trying to validate information, being aware of information-seeking gaps, having encountered challenges to gathering needed information

^a^Numbers in this column are row totals. Percentages are out of the sample of 47 participants analyzed in this paper

^b^Percentages on this row refer to the percent of the row total that falls in this column (e.g. 25% of those with low cancer worry are aware of chemoprevention)

Second, cancer worry seemed to be associated with chemoprevention awareness (Table 4). Of 24 women who fit the emergent definition of low cancer worry (thought of cancer rarely, these thoughts had minimal impact on her life), only 25% had heard of this preventive option and none were inclined to consider or use chemoprevention. Charity (African American, late 20s) knew chemoprevention was an option, but said, “Yeah…that’s not something that I would want to do. I don't even like taking Tylenol, so to me, chemo seems…drastic…” Of the 23 women who instead experienced moderate or high levels of cancer worry (thought about cancer risk at least once/month and had at least some related anxiety/fear), 65% had heard of chemoprevention, and about a third leaned toward or would consider it if recommended by a physician. Megan (White, early 40s) spent many years intensely worried about the prospect of getting breast cancer and had recently (at the time of our interview) discontinued hormone replacement therapy in spite of intense hot flashes so that she could seriously discuss chemoprevention at her next high-risk appointment.

### Choices about chemoprevention

Analyses of the content and gaps in women’s narratives about chemoprevention suggest important elements that have not previously been the focus of scientific attention. First, it was clear from women’s stories that they generally considered all the risk-management methods they knew about in tandem. Even when not in possession of a complete list or full information about options, women compared the perceived pros and cons of available choices against one another to generate personal decisions. Individual disposition toward chemoprevention explicitly reflected women’s own assessments including how high one’s risk of breast cancer is, how dangerous breast cancer is, and how drastic chemoprevention is in comparison to other options. Explicit comparisons between chemoprevention and preventive surgery were common, for instance leading some women to conclude that surgery might offer “more of a guarantee” against breast cancer, and others that “taking the preventative drugs for a few years” might be a protective stopgap until they felt more ready for preventive surgery.

The three women who used or leaned toward chemoprevention considered it an obvious choice, accepting the medication the first time it was suggested by their high-risk care provider or declaring the decision to be “a no brainer”. Of the 14 women aware of chemoprevention but disinclined to pursue it, 11 offered additional details about why. The reasons for their reluctance fell into six categories (illustrated by quotes in Table 5): (a) concerns about taking more medication or medication in general; (b) concerns about side effects or long-term health impacts; (c) feeling that chemoprevention was too drastic a measure or preferring preventive surgery for specific reasons; (d) feeling they lacked information about chemoprevention; (e) doubting the effectiveness of chemoprevention; and (f) fears that chemoprevention would have effects like chemotherapy or would negatively affect quality of life. Perhaps most interestingly, most women explained their reluctance to consider chemoprevention in terms of multiple co-existing concerns that overall rendered chemoprevention a clearly unattractive option. Nine of the 11 women who explained their reluctance expressed concerns in multiple categories; 7 expressed concerns in three or more categories. Jojo (African American, late 20s) exemplifies this exploration of many overlapping reasons to be concerned about chemoprevention:

Lately, been trying to do things in a holistic way. So, I try not to take pills, and if I do take a pill, I would like for it to be, like, a vitamin… But no, I don't think I would take a medication unless… the BRCA test came back positive… then I would maybe take the medication until I get my breasts removed or something. But no, I wouldn't want to do that because I don't like to take medicine unless I have to... It just doesn't seem…for whatever reason, surgery seems more natural to me than taking something every day because, like, when will I stop taking it? You know what I mean? If I start now, am I going to be taking it for the rest of my life? I don't know. Everyone has their preference, but my preference would be the surgery because…I wouldn't have a risk if I had the surgery. It would eliminate the risk altogether.

**Table 5:** Reasons women are reluctant to use chemoprevention

Concern	Example quotes
Taking medications, or taking additional medications (9 participants)	I don't like to take drugs. Just a personal preference. If I don't have to take pills, I don't like to take pills. (Kaitlyn, White, late 30s) I think if I weren’t on immune-suppressive therapy [taking Tamoxifen] would be more on the table. (Lucy, White, late 40s)
Known side effects or unknown potential long-term health impacts (8 participants)	The side-effects on some of those drugs are awful. (Sharon, White, late 20s) I don't always trust that this stuff is safe because if it's fairly new, sometimes you don't know for ten years. So, I guess I'd be hesitant unless there was something that was tried and true and had been out for a while. (Anne, White, late 50s)
Chemoprevention is too drastic or less definitive/proactive than preventive surgery (6 participants)	Chemoprevention seems awfully extreme. (Sharon, White, late 20s) [Surgery] is just more definitive. (Lainie, White, late 50s)
Lack of chemoprevention information (4 participants)	If I start now, am I going to be taking it for the rest of my life? I don’t know. (Jojo, African American, late 20s)
Doubts effectiveness of chemoprevention (2 participants)	It’s so unlikely to work, why put yourself through that? (Sharon, White, late 20s) From the research I’ve read, Tamoxifen is a maybe help… But it’s not like a, “You won’t get cancer if you take Tamoxifen.” (Marsha, White, early 40s)
Chemoprevention will be like chemotherapy, or will negatively affect quality of life (3 participants)	I’m hypersensitive to [medications], so the idea of taking something that might not make me feel well…is it worth quality of life? (Marsha, White, early 40s) I’d probably be a little bit more concerned [about taking Tamoxifen] because I saw my mom have such troubles with it [after she had cancer]. (Kim, White, late 30s)

Quotes taken from interviews with women who were aware of chemoprevention but disinclined or unwilling to consider it (14 out of 50 participants)

## Discussion

Low uptake of chemoprevention has been a perplexing phenomenon for breast cancer researchers, particularly given high-risk women’s expressed desire for risk-reduction options, and the considerable resources expended to develop effective chemoprevention regimens [21, 30, 48–51]. With fewer than half of these chemoprevention-eligible participants having heard of the chemoprevention option, this study highlights the importance of limited chemoprevention awareness as a key feature of low uptake. Overall, these data expose three types of knowledge gaps: general lack of chemoprevention awareness among high-risk women, particularly common lack of awareness among African Americans, and less knowledge of chemoprevention even among high-risk women aware of other risk-reduction options. These gaps are not commonly addressed in the chemoprevention literature, but pose a critical challenge to the use of chemoprevention as a risk-management method. Low levels of awareness not only constrain women’s ability to choose this risk-reduction route, they also make it impossible to assess how high-risk women would respond if they were better informed.

**Fig. 1:**
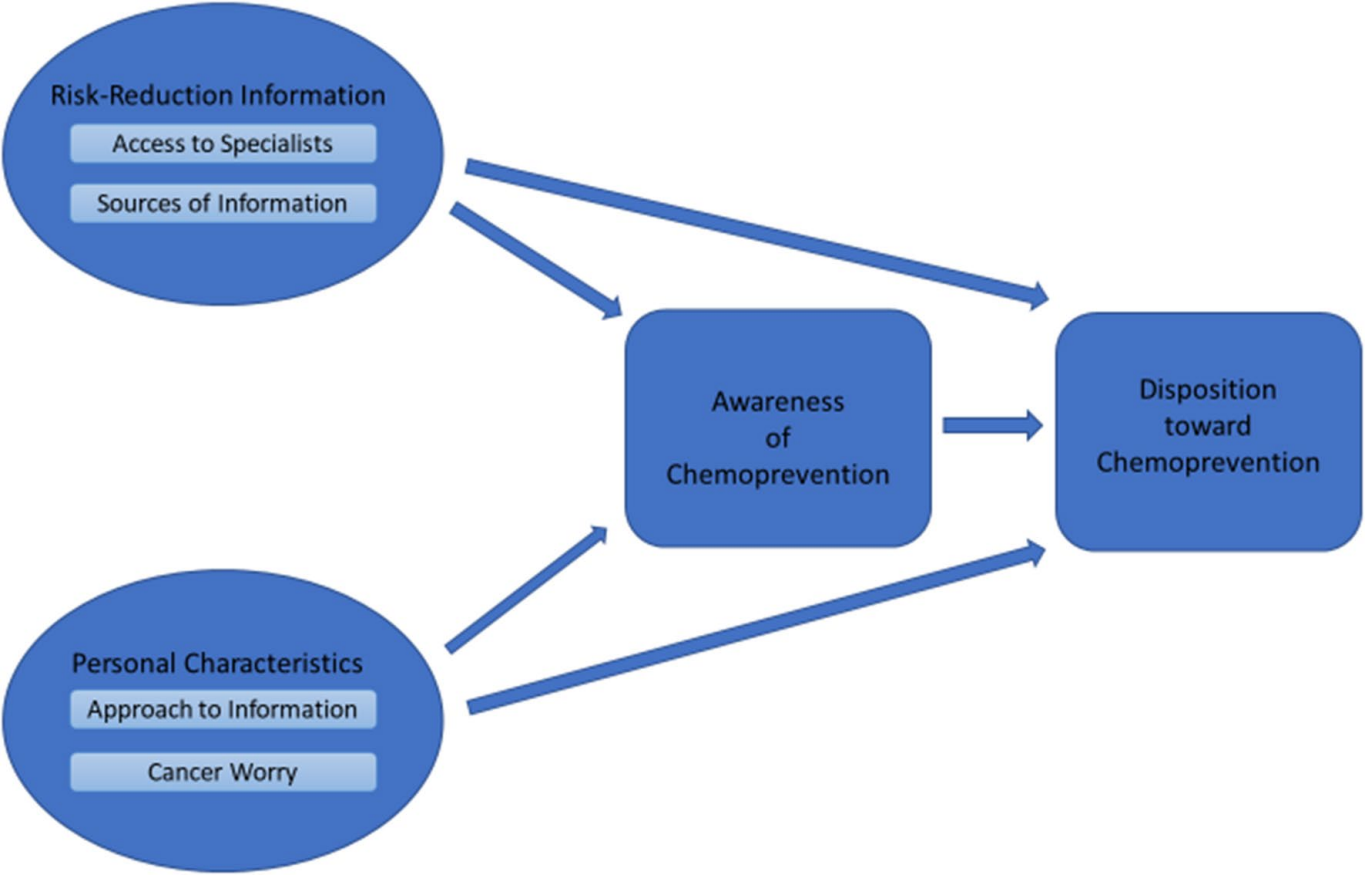
Conceptual diagram: Dynamics of considering chemoprevention

As illustrated in Fig. 1, this study suggests two categories of dynamics that may both underlie low awareness of chemoprevention and directly affect disposition toward chemoprevention among women who know about it. First, awareness of chemoprevention is likely mediated through key features of women’s environment of risk-reduction information. Most importantly, access to specialists seems to have a strong influence on awareness of chemoprevention. Our data suggest that it may only be when they see a cancer, breast, or genetics specialist that high-risk women have the dedicated risk-reduction conversations in which chemoprevention is discussed. Consistent with prior research, we find that it is largely women whose physicians recommend chemoprevention who seriously consider the option [11, 13, 14, 16–19].

Most women gather risk-reduction information from sources other than specialist health care providers. Other high-risk individuals, support groups designed for high-risk people, and family members with medical or professional expertise can be considered high-quality sources of information, in the sense that they are associated with knowing about chemoprevention. The information source most consistently likely to generate knowledge of chemoprevention as a risk-reduction option, however, seems to be conversations about risk and risk-management with any healthcare provider.

Given the important impacts of conversations with a healthcare provider on women’s attitudes toward chemoprevention, it is critical that clinicians be knowledgeable, confident, and effective in advising high-risk women about chemoprevention and other risk-reduction options. Unfortunately, recent studies document significant gaps in clinicians’ ability to provide such high-quality medical advice. The majority of healthcare providers—from generalists to breast surgeons—lack comfort and experience in identifying high-risk women using Gail or other risk assessment models [22, 52–54]. Both general practitioners and familial cancer specialists also lack core knowledge about chemoprevention and experience prescribing it [23, 55, 56]. The most common barriers physicians identify in their own ability to advise patients about chemoprevention are lack of education on the topic and lack of time to discuss it [49, 54, 55]; many also remain unconvinced that chemoprevention can be effective [22, 57] or unaware that it is clinically recommended for some groups of patients [23]. Our findings—and the additional context provided by these studies of clinician-level barriers—suggest structural interventions that could assist high-risk women in becoming fully informed about their risk-reduction options. Specifically, these could include more consistent insurance coverage for specialist consultations and genetic testing and continuing medical education programs designed to improve the ability of primary care providers to identify, advise, and refer high-risk women.

Second, these data suggest that specific personal characteristics are associated with chemoprevention awareness. In general, we find that women who use more nuanced information gathering strategies are also more likely to know about chemoprevention, and that knowing basic prevention information enables women to ask more detailed questions that could lead to actually considering specific risk-reduction behaviors [43]. Future studies should explore access to and use of risk-management information in more detail, since these dynamics are critical to the ability of high-risk women to make health-protective decisions. In addition, while the causal direction of the relationship between higher cancer worry and higher awareness of chemoprevention could hypothetically go either way, this general association again suggests that it might be useful to educate and enable primary care providers to discuss risk-related information with *all* high-risk patients.

Finally, this study and others indicate that women’s concerns about chemoprevention are quite complex [15, 20, 58]. The few women in this study who had taken a chemoprevention regimen retrospectively assessed the decision as an obvious and clear-cut choice to dramatically reduce their breast cancer risk. The substantial proportion of women who knew about chemoprevention but were not inclined to pursue it, however, articulated a range of overlapping reasons for their reluctance, from lack of information (about side effects, long-term health risks, or effectiveness) to semi-confident preferences (to avoid medication or pursue alternate risk-reduction approaches). The reluctance to use any medication at all (see also [32, 33]) is not generally consistent with the use of medication in the U.S. overall, or with the far more substantial uptake of other preventive medications (such as statins). Furthermore, women’s assessment of chemoprevention as more drastic than prophylactic surgery and potentially having very acute and serious side effects suggests that perhaps women fear chemotherapy-like experiences while on chemoprevention, despite rare evidence that this occurs [58, 59]. If this is the case, it might have appeared in our data in the form of (a) explicit references to chemotherapy in women’s discussions of chemoprevention, and (b) associations between women reluctant to use chemoprevention and those who knew someone who had been on chemotherapy. Our data did not contain these patterns, but larger samples could be used to investigate this possibility more systematically. The terms used to describe chemoprevention may be critical—we used “medication that can reduce your risk of cancer” unless participants already knew the term “chemoprevention”, but it would be worth investigating the frequency with which the physicians who educate high-risk women use “chemoprevention” vs. the names of specific drugs or general descriptors like ours. Overall, the complex patterns surrounding women’s concerns both deepen the mysteries surrounding low chemoprevention uptake and suggest further research directions that could clarify the issue.

The main limitation of this study has to do with its sample. Forty-seven in-depth interviews is an appropriate number for inductive research seeking deep understanding of women’s experiences, and met the primary sample-size criterion of achieving theoretical saturation. A larger sample size of high-risk women—recruited to represent the broader population and not just those in high-risk care or in a chemoprevention trial—would allow confirmation of these findings, as well as quantitative analysis of predictors of chemoprevention awareness and disposition. In addition, it would be helpful for future samples to include women who are high-risk not only because of familial history but also due to personal history of atypical hyperplasia or lobular carcinoma in situ, since these patients are particularly likely to benefit from chemoprevention. Finally, some important outstanding questions can only be addressed with a larger sample, such as about the mechanisms that drive racial and SES disparities in chemoprevention awareness.

## Conclusions

The results presented here illuminate previously unaddressed complexities in chemoprevention decision making. First, although most risk-reduction *studies* focus on each option separately, *women* do not, so incomplete information about risk-prevention methods or their consequences artificially constrains women’s choices. Second, the women in this sample do not simply use or reject chemoprevention; their opinions instead exist on a continuum of dispositions that could change with age, new information about hereditary risk, or physician advice. Taking a dispositional approach to prevention decisions, as some decision-aid developers have begun to do, may help us more thoroughly understand and support women’s decisions. Third, most women voice not one but multiple reasons they are reluctant to take antiestrogen medications. Some of these reasons reflect information gaps that could be filled to facilitate informed decision making, while others likely reflect more stable preferences of informed women.

These findings point in new scientific directions, but also have important clinical implications. It is essential that high-risk women be informed of all their risk-reduction options—and it is clear that this is not currently the case. Filling the critical gap in chemoprevention awareness will require improvements in healthcare and health systems serving this population. Primary care providers may play an integral role, by identifying high-risk women and giving them basic information about risk and prevention [34, 35], pointing women toward patient-information libraries and decision aids that may aid in self-education [15, 34, 35], and referring them to specialists who can discuss individual risk and risk-management options in detail. Given the formative role of specialist access, ensuring that women have health insurance and that their plans cover specialist care and genetic testing is also crucial. Finally, future attempts to facilitate informed risk-management decision-making will require closer attention to the nuances of women’s disposition toward their options and the multiple factors they consider when making risk-management decisions.

## Supplementary Information

**Additional file 1.** Contains the interview protocol through which data for the parent project was collected.

## Data Availability

The datasets generated and/or analyzed during the current study are not publicly available due confidentiality but are available from the corresponding author on reasonable request.

## Abbreviation

SES: Socioeconomic status
