# Correction to: Understanding low chemoprevention uptake by women at high risk of breast cancer: findings from a qualitative inductive study of women’s risk-reduction experiences

**DOI:** 10.1186/s12905-021-01351-z

**Published:** 2021-05-17

**Authors:** Tasleem J. Padamsee, Megan Hils, Anna Muraveva

**Affiliations:** 1 Division of Health Services Management and Policy, College of Public Health, The Ohio State University, 280F Cunz Hall, 1841 Neil Avenue, Columbus, OH 43210 USA; 2 Lutheran Social Services of Central Ohio, Worthington, OH USA; 3 Division of Health Services Management and Policy, College of Public Health, The Ohio State University, Columbus, OH USA

## Correction to: BMC Women’s Health (2021) 21:157 10.1186/s12905-021-01279-4

Following the publication of the original article [1], we have been notified of a few alignment issues in Table 3.

The original article has been corrected.

1. Padamsee TJ, et al. (2021) Understanding low chemoprevention uptake by women at high risk of breast cancer: findings from a qualitative inductive study of women’s risk-reduction experiences. BMC Women's Health (2021) 21:157. https://doi.org/10.1186/s12905-021-01279-4

