# Supplementary material for: Understanding low chemoprevention uptake by women at high risk of breast cancer: findings from a qualitative inductive study of women’s risk-reduction experiences
Source: BMC Womens Health. 2021 Apr 16;21:157. doi: 10.1186/s12905-021-01279-4 (PMC8052843; doi:10.1186/s12905-021-01279-4)
Supplement: Supplementary file 1 — Additional file 1. Contains the interview protocol through which data for the parent project was collected. [file 12905_2021_1279_MOESM1_ESM.docx]

**Supplementary File – Interview Protocol**

This research is based on a parent project that included fifty in-depth, semi-structured interviews with a purposive sample of African American and White women at elevated risk of breast cancer. This specific study draws on the forty-seven interviews conducted with women at high or severe risk of breast cancer, all of whom are eligible to use chemoprevention for breast cancer risk reduction. The semi-structured interview protocol below was used to guide interview data collection.

**Initial Questions***Starter questions were asked as needed. Participants were encouraged to tell their own stories as fully as possible, using the non-guiding probes listed to keep the story moving and fill in gaps.*

1. When and how did you discover that you were at particular risk for breast cancer?
2. Could you tell me about any history of breast cancer in your family?
3. What kinds of actions have you considered to prevent breast cancer in your future?
4. What decisions have you made? What decisions are you still making, or do you plan to make at some point?
5. How did you make these decisions?
6. Who did/do you talk to about your risk or decisions?
7. What were/are those conversations like?
8. How have/do you feel about your breast cancer risk, and your prevention decisions?
9. How much/what kind of an impact have your breast cancer risk and prevention decisions had on your life?
10. What has helped you in dealing with breast cancer risk and prevention?
11. What might have helped you deal better with it, or feel better about it?

**Non-Guiding Probes**

- What happened next?
- How did/do you feel about that *[realization of elevated risk, particular conversations or deliberations, current status]*?
- Can you tell me some more detail about X?

**Follow-Up Questions***Once participants told their initial stories, detailed questions and probes (presented in abbreviated form here) were used in each of the following categories to elicit details as needed, until each area had been thoroughly discussed.*

1. Risk Status
   - What is your individual level of risk for breast cancer in the future?
   - What have you been told about this?
   - Why do you have this particular level of risk?
2. Sources and Content of Risk Information
   - Where, and from whom, have you gotten information about your risk of breast cancer? Include discussion of family members, friends, medical professionals, support organizations, other research
   - How did you come to your current understanding of your own risk level?
3. Prevention Options

- Have you considered doing anything to decrease your chances of having breast cancer in the future?
- What have you considered doing?
- Topics covered: prophylactic oophorectomy, prophylactic mastectomy, chemoprevention, additional screening, other

1. Decision-Making Process

- Where did you get information about the possibility of X prevention option (mastectomy, oophorectomy, chemoprevention, enhanced surveillance, nutrition, alternative therapies, other)?
- How did you think through (or how are you thinking through) your prevention options?
- Include where the idea first came from, whom they talked to about it, feelings about options or decisions, critical turning points in decision making, information and support both present and missing.

1. Decision-Making Network

- Who has contributed to your prevention decision making (include medical professionals, family members, friends, support groups, community organizations, other)?
- How have they contributed to your decision making (include types of conversations, content, what was useful and not useful, impact of conversations on decision making)?

1. Psychosocial Well-being

- What emotions have you experienced while dealing with breast cancer risk and prevention?
- Which emotions have been dominant for you (explore anxiety, worry, fear, stress, depression, body image, desire for control of health, concerns about sexuality, cancer-related stigma, self-assessment of health)?
- How much impact – and what types of impacts – have these feelings had on your breast cancer prevention decisions, relationships, life more generally?

1. Resources

- How much of your time and energy has decision making and prevention activity consumed (cover different points in time)?
- How much has your prevention activity cost you financially (cover information gathering, healthcare, appointments, screening procedures, prevention activities, role of health insurance)?
- To what degree has financial cost influenced your thoughts or decisions about prevention options?
